# Shared reading quality assessment by parental report: preliminary validation of the DialogPR

**DOI:** 10.1186/s12887-018-1298-1

**Published:** 2018-10-18

**Authors:** John S Hutton, Guixia Huang, Kieran J Phelan, Thomas DeWitt, Richard F Ittenbach

**Affiliations:** 1 Division of General and Community Pediatrics, Cincinnati Children’s Hospital Medical Center, 3333 Burnet Avenue, MLC 7035, Cincinnati, OH 45229 USA; 2 Reading and Literacy Discovery Center, Cincinnati, USA; 3 Division of Biostatistics and Epidemiology, Cincinnati Children’s Hospital Medical Center, Cincinnati, OH USA

**Keywords:** Reading/literacy, Family dynamics/processes, Mother-child relations, Dialogic reading, Shared reading, Home literacy environment, Measure development, Assessment/testing

## Abstract

**Background:**

The American Academy of Pediatrics (AAP) recommends shared reading beginning as soon as possible after birth to promote healthy development. Shared reading quality can strongly influence outcomes, especially in children from low-SES households. Dialogic reading is a method developed to enhance verbal interactivity and engagement through book sharing, advocated by the AAP and clinic-based programs such as Reach Out and Read. There is no brief, validated, caregiver report measure of dialogic reading or shared reading quality currently available.

**Methods:**

This cross-sectional study involved 49 healthy mother-child dyads (mean child age 4.5 yrs., *SD* = 0.6 yrs.) from 2 separate MRI-based studies. The DialogPR was administered by trained research coordinators following MRI, along with the READ subscale of the validated StimQ-P measure of home cognitive environment. The DialogPR consists of eight items developed in consultation with experts in early literacy, based on the PEER/CROWD dialogic reading conceptual model. Estimated reading level is 6th grade. Descriptive statistics were computed at both the item and scale levels. Modern theory Rasch methods were used to analyze all eight DialogPR items along with preliminary estimates of reliability and validity.

**Results:**

Our combined sample involved 15 boys and 34 girls, and was diverse in terms of age, household income, and maternal education. DialogPR administration time was less than 2 min, with no problems reported. The DialogPR demonstrated strong internal consistency and reliability (Cronbach’s alpha = 0.82), and criterion-related validity with the StimQ-P READ (Spearman’s rho coefficient = 0.53). Rasch analysis revealed strong psychometric properties in terms of reliability, variability in item difficulty, and inter-item and item-measure correlations.

**Conclusions:**

Preliminary evidence suggests that the DialogPR may be an efficient means to assess shared reading quality and dialogic reading via caregiver report for clinical and research purposes, warranting further investigation.

## Background

The American Academy of Pediatrics (AAP) recommends literacy promotion in primary care beginning as soon as possible after birth [1]. Literacy is a major social and public health issue, the cost of low achievement estimated at over $350 billion per year in the United States, and over $1.2 trillion worldwide [2]. As of 2015, 64% of US 4th graders scored below proficient in reading, largely unchanged from prior reports [3], and lower still for children from minority and low-socioeconomic status (SES) households [3, 4]. While 5 to 14% of reading difficulties have an organic cause (e.g. dyslexia) [5], the majority are environmentally based and largely preventable, a consequence of inadequate resources, motivation and/or stimulation required to learn to read [2]. Many children arrive at school at a substantial disadvantage in readiness, unlikely to catch up with peers as academic demands accelerate [4]. Thus, early screening and intervention offer large potential savings in terms of productivity and health [6–8].

Parents are considered to be a child’s “first and most important teachers [9].” Cognitive stimulation in the home, exemplified by shared reading [10–12], greatly influences educational and health outcomes [13, 14]. Literacy promotion programs based in pediatric clinics (notably Reach Out and Read [15]), preschool [16], and home visitation [17] share a goal of enhancing home literacy environment, a composite of quantitative and qualitative factors [1, 18]. Quantitative factors typically include number of children’s books in the home, frequency of shared reading (e.g. days per week, minutes per day), approximate age when shared reading was initiated, and variety/type of books read [19, 20]. Qualitative factors typically include parent and child interest in and enjoyment of reading, and verbal and social-emotional interactivity during shared reading. Given the ease of assessment via parental report, quantitative factors are most often screened and addressed [21–23]. However, qualitative factors such as verbal interactivity during shared reading may be even more important and have significant impact [14, 24], though are often overlooked.

Dialogic reading is a method of shared (usually parent-child) reading developed to promote reciprocal dialogue between a caregiver and child during story sharing [25], advocated by Reach Out and Read and the AAP [1, 15]. The acronym PEER is used to reflect the dialogic process [26], as follows: 1) Prompt the child to say something about the story, 2) Evaluate what the child says, 3) Expand on what the child says, and 4) Repeat and reinforce associations. Similarly, the acronym CROWD is used to reflect evocative caregiver prompts: 1) Completion (of a sentence), 2) Recall earlier aspects of the story, 3) Open-ended questions, 4) *Wh-* questions, and 5) Distancing (relate the story to the child’s experience). Behavioral evidence suggests that this qualitative aspect of shared reading may confer moderate to large cognitive and social-emotional benefits beginning in infancy [27], especially for children from low-SES backgrounds [28, 29]. However, there is currently no validated measure of dialogic reading or shared reading quality currently available that is feasible for clinical use.

The purpose of this study was to develop and pilot test a brief caregiver report measure of shared reading quality (DialogPR) based on a dialogic reading conceptual model. Our eight-item measure was reviewed by experts in measure design and child development, pilot tested for clarity, and then administered as an exploratory aim in 2 unrelated, MRI-based studies involving healthy, preschool-age children and their mothers: one comprised exclusively of low-SES (*n = 22*) and the other of largely higher-SES (*n = 27*) dyads. The validated StimQ-P measure of cognitive stimulation in the home [30, 31], which includes a subscale of home reading practices, was administered as an external standard. Psychometric analyses, including modern-theory Rasch modeling, were performed. Our hypothesis was that the DialogPR would be feasible to administer, reliable, and valid in this combined sample, attesting to the value of a cohesive conceptual model of dialogic reading, warranting further investigation.

## Methods

### Sample

This study involved 49 healthy mother-child dyads enrolled in two recent MRI-based studies of cognitive and brain development at our institution, which were combined for the present analysis. Inclusion criteria for both studies were: preschool-age (3–5 years), full-term gestation, native English-speaking household, no history of brain injury, developmental delay or stimulant use, and no contraindications to MRI. The first sample (*n* = 22) was drawn from an ongoing home-injury prevention trial involving low-SES families at-risk for poor health and social outcomes [32]. Girls were exclusively sampled for this study due to time/budget constraints and higher MRI success rates for girls at this age [33]. The second sample (*n* = 27) involved mother-child dyads recruited via advertisement from employee families at a large academic medical center, with no gender constraint [34]. Families were compensated for time and travel, and each study was approved by the Cincinnati Children’s Hospital Institutional Review Board.

### Instrument

The conceptual model for our DialogPR instrument was the PEER/CROWD dialogic reading construct developed by Whitehurst, et al. [25]. Our DialogPR instrument included a scripted introduction: “*When deciding your answer, try to think about how you and [CHILD’S FIRST NAME] have read children’s books together over the past month.”* It was comprised of eight questions: 1) frequency of discussing what the book might be about before reading, 2) frequency of discussion during story sharing, 3) five questions referencing frequency of respective CROWD prompts during story sharing, and 4) frequency of discussion of what the book was about after reading. Categorical responses for questions 1, 2, and 8 were: “always,” “usually,” “sometimes,” and “never.” Responses for CROWD questions 3 to 7 were anchored to a hypothetical book (“*When you are reading a children’s book with [CHILD’S FIRST NAME], how often do you stop reading to do the following things?*”): “on most pages,” “on around half of the pages,” “on a few pages,” or “never.” An ordinal scoring system was used for each question, with response options ranging from 0 to 3 points for each item, with higher scores reflecting greater frequency of dialogic behaviors.

Wording for the DialogPR was refined in consultation with experts in measure development at our institution, and pilot tested for clarity among colleagues and families attending a hospital-based primary care clinic. Estimated Flesch-Kincaid reading level was 6th grade. Research coordinators practiced administration with the principal investigator and were instructed to adhere to instrument wording verbatim. Research coordinators administered the DialogPR to mothers at the study visit following the child’s MRI, and transcribed response data into a secure REDCap database [35].

### Reference measure

The StimQ-P served as the criterion-referenced standard for this study [30, 31], and was administered to mothers following the DialogPR. The StimQ-P is a validated parental report measure of cognitive stimulation in the home for children 36 to 72 months of age, and consists of 4 subscales involving mostly “yes/no” questions: 1) Availability of learning materials (ALM); 2) Reading (READ), reflecting access to books, frequency of shared reading, variety of books read, and interactivity/quality of reading; 3) Parental Involvement in Developmental Advance (PIDA); and 4) Parental Verbal Responsivity (PVR). StimQ has been found to have excellent internal consistency (Cronbach’s alpha 0.88 to 0.93), convergent validity with the HOME inventory, and predictive/concurrent validity with language scores, including for its subscales [30]. For the aims of this study, and to keep our assessment brief, only the READ subscale was administered.’

### Statistical analyses

Data analysis proceeded in three distinct steps. First, demographic characteristics were computed for the entire sample of 49 children. Second, descriptive statistics were computed for all variables in the data set, at both the scale and item levels. All eight DialogPR items were evaluated for smoothness, modality, difficulty, polarity, and sufficiency of observations across levels. Modern theory Rasch rating scale methods were used for analysis due to the identical, ordered categorical nature of response options across all items [36, 37]. Model fit was tested for each item to identify any that were markedly or unnecessarily influencing the scale-level distributions. Third, preliminary estimates of DialogPR’s reliability and validity were computed, beginning with Cronbach’s coefficient alpha (α_Cr_) and standard error of measurement (SEM) as our measures of reliability, and a Spearman-rho (*r*_ρ_) correlation coefficient between DialogPR total score and StimQ-P READ subscale score as our measure of criterion-related validity. Spearman-rho correlation was chosen given relatively small sample size warranting a conservative, non-parametric approach. The criterion for statistical significance was set at the unadjusted α = 0.05 level due to the preliminary nature of the study. All analyses were conducted using SAS v9.4 and Winsteps v4.0 software.

## Results

### Demographic characteristics

This study involved two sample populations from separate studies involving mothers and their preschool-age children. These were combined in the present analyses, and are summarized in Table 1.

**Table 1:** Demographic Characteristics of Participants (*n* = 49)

Variable	*n* (%)
*Child Gender*	
Female	34 (69.4)
Male	15 (30.6)
*Child Age Group*	
3 to 4 Years	9 (18.4)
4 to 5 Years	26 (53.0)
5 to 6 Years	14 (28.6)
*Household Income*	
Less than $15,000	17 (34.7)
$15,000 to $50,000	6 (12.2)
$51,000 to $99,000	12 (24.5)
More than $100,000	14 (28.6)
*Maternal Education Level*	
High School Diploma/GED or Less	13 (26.5)
Some College	13 (26.5)
Bachelor’s/Four Year Degree	15 (30.6)
Graduate/Professional Education	8 (16.3)

### Descriptive statistics for the DialogPR

**Fig. 1:**
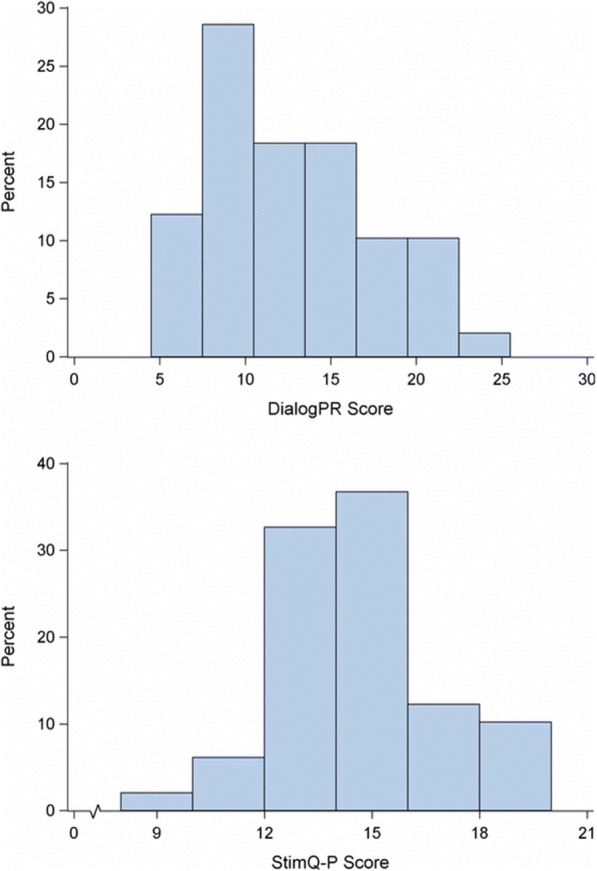
Distributions of DialogPR and StimQ-P READ Scores. Histograms for DialogPR and StimQ-P total scores (*n* = 49). Shapiro-Wilk tests revealed non-normality for each (*p* = 0.04 for each), and non-parametric analyses were conducted

Research coordinators reported no difficulty administering the DialogPR, with all subjects completing the survey in less than 2 min. Mean DialogPR score was 12.6 (*SD* = 4.8), and ranged from a minimum of 5 to a maximum of 24. Mean STIMQ-READ score was 14.1 (*SD* = 2.4) and ranged from a minimum of 9 to a maximum of 19 across 49 participants. Histograms of DialogPR and StimQ READ score distributions are provided in Fig. 1.

### Item analysis

Item level information for the DialogPR is provided in Table 2. Rasch estimates of item difficulty ranged from − 2.00 (less difficult) to 1.06 (more difficult). Point-measure correlations ranged from 0.44 (item 2) to 0.73 (item 4), suggesting a moderate relationship between each of the DialogPR items and the entire scale. Item fit statistics using empirically-derived *z*-values were all well below the traditional + 2 standard deviations, suggesting no outliers likely to influence the distributions [38]. With respect to inter-item correlations of the DialogPR, significant correlations ranged from *r*_ρ_ = 0.29 (Q2-Q6; low) to 0.62 (Q3-Q4; moderate), shown in Table 3. There was no significant correlation between child gender (male = 1) and DialogPR item or total scores, with the exception if item 2, which was negatively correlated (*p* < 0.05). Household income was negatively correlated with items 2, 4, 5, 6, and total DialogPR score (*p* < 0.05). Maternal education level was negatively correlated with item 6 only (*p* < 0.05). These demographic correlations are summarized in Table 3. StimQ-P READ score was negatively correlated with household income and maternal education (*p* < 0.05), but not with child gender.

**Table 2:** Item Analysis and Summary Statistics for DialogPR (Rasch Analysis)

Item	M(SD)	Difficulty	Standard Error	Infit z	Outfit z	Point-Measure Correlation
Recall (Q4)	1.1 (0.9)	1.06	0.23	0	− 0.5	0.73
Discuss Before (Q1)	1.1 (0.9)	1.01	0.22	−1.2	−1.8	0.72
Completion (Q3)	1.1 (0.9)	1.01	0.22	1.0	0.7	0.63
Distancing (Q7)	1.2 (0.8)	0.86	0.22	0.2	0.2	0.62
Open-ended (Q5)	1.8 (1.0)	−0.47	0.21	−0.2	0.7	0.72
“Wh” Questions (Q6)	1.8 (0.9)	−0.60	0.21	−1.3	−1.6	0.71
Discuss After (Q8)	2.0 (1.0)	−0.86	0.21	0.9	0.4	0.64
Respond (Q2)	2.4 (0.7)	−2.00	0.23	1.8	0.5	0.44

Note. Terms used here to describe items are summaries of the main concepts of the items themselves. Actual items numbers are abbreviated as Q1 through Q8

Rasch and item-level summary statistics for DialogPR scores (*n* = 49), including mean (M), standard deviation (SD), Difficulty, standard error, internal fit, external fit, and Point-Measure (item-total score) correlation. Terms referenced with each item number briefly summarize the main purpose of the item. Items are presented in order of difficulty, from most difficult (1.06) to least difficult (− 2.00)

**Table 3:** Intercorrelation Table for DialogPR Items, StimQ-P READ Score and Selected Demographic Characteristics

	Q2	Q3	Q4	Q5	Q6	S7	Q8	Dialog PR Total Score	StimQ-P READ Score	Child Gender	Household Income	Maternal Education
Q1	0.22	0.50^*^	0.39^*^	0.28	0.48^*^	0.27	0.54^*^	0.67^*^	0.24	0.04	−0.11	−0.10
Q2		− 0.07	0.18	0.43^*^	0.29^*^	0.13	0.17	0.42^*^	0.08	−0.29^*^	−0.49^*^	− 0.24
Q3			0.62^*^	0.24	0.26	0.26	0.25	0.57^*^	0.25	0.13	0.04	0.02
Q4				0.49^*^	0.34^*^	0.48^*^	0.24	0.69^*^	0.41^*^	−0.05	−0.31^*^	−0.25
Q5					0.56^*^	0.39^*^	0.42^*^	0.76^*^	0.44^*^	−0.16	−0.35^*^	−0.15
Q6						0.35^*^	0.49^*^	0.72^*^	0.48^*^	−0.21	−0.37^*^	−0.34^*^
Q7							0.39^*^	0.62^*^	0.47^*^	−0.04	−0.24	−0.15
Q8								0.69^*^	0.40^*^	−0.01	−0.14	−0.08
Total Score									0.53^*^	−0.09	−0.36^*^	−0.24

Spearman-rho intercorrelation coefficients between DialogPR items 1 through 8 and total score, and with StimQ-P READ total score, child female gender, household income, and maternal education level (*n* = 49). * denotes significant correlations (*p* < 0.05)

### Reliability and validity

For reliability, internal consistency was acceptable to good at *r*_*Co-*α =_ 0.82. The DialogPR’s standard error of measurement, a measure of reproducibility of test scores, was estimated at SEM = 2.0. This means that the DialogPR “true” score for participants is expected to fall, on average, within the range of 8.7 to 16.5, 95% of the time. For criterion-related validity, the correlation between the DialogPR and the STIMQ-READ subscale score was *r*_ρ_ = 0.53 (*p* < 0.001). There was significant, positive correlation between individual DialogPR items and StimQ-READ (*p* < 0.05) with the exception on items Q1, Q2, and Q3, which were non-significant (Table 3).

## Discussion

Literacy is a major predictor of educational, occupational and health outcomes [2]. While causality has not definitively been proven [39], important drivers of reading difficulties include deficient or absent reading role models in the home and consequently impaired abilities, attitudes and routines [40, 41]. Substantial resources are devoted to initiatives to enhance home literacy environment [14, 15, 42], efficacy and improvement dependent on the ability to collect data efficiently and reliably. Research to date has largely relied on aspects of home literacy environment that are straightforward to assess via parental report, such as access to books and reading frequency, potentially neglecting critical behaviors such as verbal interactivity. These “dialogic” qualities may be particularly at-risk in parents lacking confidence or experience with shared reading, such as those from low-SES households [43], and can influence reading outcomes [24, 28].

The DialogPR instrument performed remarkably well in preliminary psychometric analysis. With only 8 items and brief administration time, this performance suggests potential value in clinical and research settings, though more expansive validation studies are needed. We attribute this strong performance to an evidence-based conceptual model of dialogic reading [26], which guided item development. In general, DialogPR items showed low to moderate inter-item correlation and good response variability, suggesting that each contributed uniquely to the overall score, and that parents could identify their own, shared reading behavior in a range of options. The five items corresponding to specific CROWD prompts (items 3–7) each performed well, with moderate correlations between them suggesting cohesion as reading behaviors. Interestingly, items 3 and 4 were less strongly correlated with the other CROWD items than with each other, which we suspect may be attributable to completion and recall prompts seeming more abstract or unnatural to some parents at this age, compared to the other types of questioning. This may also be why items 3 and 4 were the behaviors parents reported least often. By contrast, more straightforward items 5 and 6 (open-ended and *wh-* questions) were more strongly correlated with each other than with the other CROWD items, yet were among the easiest to endorse. Items 1 and 8, reflecting discussion before and after reading, respectively, and not core PEER/CROWD components, were also among the most highly correlated item pairs, yet discussion before reading was reported far less frequently.

The item performing the weakest (though still respectably), item 2, concerns frequency of pauses to answer a child’s questions, intended to reflect dialogic evaluation and expansion (Es in PEER). Possibly due to its general wording, all but 7 parents responded that they “always” or “usually” do so, the other 7 responding “sometimes,” and none responding “never.” It is intriguing that the only significant inter-item correlations involving item 2 were with items 5 (open-ended questions) and 6 (“*wh-*” questions). While speculative, this finding seems intuitive and suggests that this sample of mothers associated these types of relatively straightforward prompts with further dialogue. Variability in this item may have been higher if it instead asked how often specific types of responses (e.g. evaluations and expansions) were made. Such refinement in future versions of DialogPR to more explicitly assess evaluation and expansion during shared reading, may be worthwhile.

Initial evidence suggests that the most frequently endorsed items for these parents, reflecting the most common or least “difficult” shared reading behaviors, were item 2 (frequency of pauses during reading to respond to a child’s questions or comments, discussed above), item 8 (discussion after reading), item 6 (“*wh-*” questions), and item 5 (open-ended questions). The least commonly endorsed, or most “difficult” shared reading behaviors were item 4 (recall prompts), item 1 (discussion before reading), and item 3 (completion prompts). Overall, this item performance seems highly intuitive, the more straightforward, or “natural” behaviors reported most often, with the possible exception of discussion before reading to generate interest. Interestingly, this finding also concurs with shared reading observations conducted for a separate MRI study involving our low-SES population (*n* = 22) [32] where *wh-* prompts and open-ended questions were used almost exclusively.

Variability in item responses (with the marginal exception of item 2), and even slight skew towards lower scores, suggests that the DialogPR was not overly influenced by social desirability bias, a universal concern in parental report measures [44]. While both DialogPR and StimQ READ scores were not technically normally distributed (Shapiro-Wilk test, *p* = 0.04), strong correlation between DialogPR and this validated instrument is also reassuring in this respect. Interestingly, DialogPR total scores were negatively correlated with household income, though not with child gender or maternal education. StimQ-P READ scores were similarly negatively correlated with income, and also with maternal education. While speculative, these paradoxical findings may be attributable to a more nuanced type of reporting bias, where mothers from low-SES backgrounds may be more likely to over-report desirable reading behaviors, particularly ones that are more straightforward (in this sample, notably ‘*wh-‘*questions and responding to the child), while mothers from higher-SES backgrounds may be more critical of their reading behaviors. Comparison between DialogPR scores and direct observation of shared reading in the home would be useful to quantify potential reporting effects, though this may be difficult in practice with a large sample.

This study has several important strengths. The DialogPR was developed and refined referencing an evidence-based conceptual model of dialogic reading, which afforded clarity in item content and organization. Despite a small sample size, it exhibited remarkably strong psychometric properties using advanced analytic modeling techniques. DialogPR scores were highly correlated with a validated parental report measure of reading behaviors in the home (StimQ-P READ). Administration time was brief with no concerns reported in two samples of parents from diverse backgrounds, including low-SES families who are in greatest need of effective assessment and intervention. The DialogPR addresses an important evidence gap and need for an efficient assessment of shared reading quality via caregiver report, with potential research and clinical application in programs advocating dialogic reading such as Reach Out and Read.

Our study also has a number of limitations that should be noted. Our sample population was a combination from 2 smaller studies, which can also be viewed as a strength, and efficient, innovative use of resources. The first exclusively involved 4-year-old girls from low-SES households by design, while the second was diverse in age (3 to 5) and gender from largely higher-SES employee families at an academic medical center. Together, this provided a remarkably diverse sample, showing that DialogPR may be generalizable, though a larger, inherently diverse sample would help affirm this. Girls are marginally over-represented, though it is reasonable to assume that shared reading quality should not be overly dependent on a child’s gender, especially in the preschool age range. For budgetary reasons, we were not able to conduct follow-up visits, and thus unable to assess test-retest reliability. We were unable to compare directly these findings to direct observation, and it is unclear whether reported behaviors reflect a long-term pattern, especially in oft-chaotic home environments. Most importantly, while this study offers a respectable first step, our relatively small sample size is inadequate for definitive validation, and future research with a larger sample will be needed to corroborate the findings here, including test-retest reliability and, ideally, concurrent validity with observational data. Further refinement is needed, perhaps including more explicit means to assess parental responses to the child (i.e. evaluations and expansions) during shared reading, in a parsimonious way. Overall, at this preliminary stage, the DialogPR offers a conceptually- and psychometrically-sound step toward improved, efficient insight into dialogic reading and shared reading quality in the home, important catalysts for healthy cognitive- and social-emotional development.

## Conclusion

In this pilot study involving a relatively small (*n* = 49) yet diverse sample of mothers of preschool-age children, the 8-item DialogPR exhibited strong and promising psychometric properties, including internal consistency, reliability and validity referenced to an external standard of home literacy environment. The DialogPR is founded on a conceptual model of dialogic reading, which is advocated by the AAP and programs such as Reach Out and Read to improve verbal interactivity and engagement through book sharing, and may be an efficient, valid means of assessment warranting further investigation.

## Abbreviations

AAP: American Academy of Pediatrics
MRI: Magnetic resonance imaging
SES: Socioeconomic status
